# 2-Phenyl-4-(3,4,5-trimethoxy­benzyl­idene)-1,3-oxazol-5(4*H*)-one

**DOI:** 10.1107/S1600536808005746

**Published:** 2008-03-07

**Authors:** Yi-Feng Sun, Yi-Ping Cui

**Affiliations:** a Advanced Photonics Center, School of Electronic Science and Engineering, Southeast University, 210096 Nanjing, Jiangsu, People’s Republic of China; b Department of Chemistry, Taishan University, 271021 Taian, Shandong, People’s Republic of China

## Abstract

The title compound, C_19_H_17_NO_5_, was synthesized as part of a continuing project involving the structures of oxazolone derivatives. The mol­ecule adopts a *Z* configuration about the central olefinic bond. The 2-phenyl ring is slightly twisted out of the plane of the oxazolone ring system by 11.2 (2)°. The crystal structure is stabilized by weak inter­molecular C—H⋯O hydrogen bonds.

## Related literature

For background literature, see: Aaglawe *et al.* (2003 ▶); Grassi *et al.* (2004 ▶); Khan *et al.* (2006 ▶); Song *et al.* (2001 ▶). For related structures, see: Sun *et al.* (2007 ▶); Imhof & Garms (2005 ▶); Song *et al.* (2004 ▶); Vasuki *et al.* (2001 ▶).
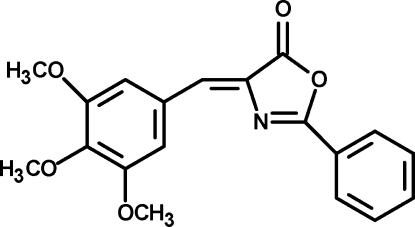


## Experimental

### 

#### Crystal data

C_19_H_17_NO_5_
*M* *_r_* = 339.34
Triclinic, 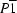

*a* = 7.3897 (5) Å
*b* = 8.1532 (6) Å
*c* = 14.0023 (9) Å
α = 86.917 (5)°
β = 83.306 (4)°
γ = 82.471 (5)°
*V* = 830.02 (10) Å^3^
*Z* = 2
Mo *K*α radiation
μ = 0.10 mm^−1^
*T* = 273 (2) K
0.15 × 0.12 × 0.10 mm

#### Data collection

Bruker SMART CCD area-detector diffractometer
Absorption correction: multi-scan (*SADABS*; Sheldrick, 1996 ▶) *T*_min_ = 0.985, *T*_max_ = 0.990
4665 measured reflections
2904 independent reflections
2056 reflections with *I* > 2σ(*I*)
*R*_int_ = 0.020

#### Refinement

*R*[*F* ^2^ > 2σ(*F* ^2^)] = 0.043
*wR*(*F* ^2^) = 0.121
*S* = 1.04
2904 reflections
229 parameters
H-atom parameters constrained
Δρ_max_ = 0.12 e Å^−3^
Δρ_min_ = −0.15 e Å^−3^

### 

Data collection: *SMART* (Bruker, 1997 ▶); cell refinement: *SAINT* (Bruker, 1997 ▶); data reduction: *SAINT*; program(s) used to solve structure: *SHELXS97* (Sheldrick, 2008 ▶); program(s) used to refine structure: *SHELXL97* (Sheldrick, 2008 ▶); molecular graphics: *SHELXTL* (Sheldrick, 2008 ▶); software used to prepare material for publication: *SHELXTL*.

## Supplementary Material

Crystal structure: contains datablocks global, I. DOI: 10.1107/S1600536808005746/pk2085sup1.cif

Structure factors: contains datablocks I. DOI: 10.1107/S1600536808005746/pk2085Isup2.hkl

Additional supplementary materials:  crystallographic information; 3D view; checkCIF report

## Figures and Tables

**Table 1:** Hydrogen-bond geometry (Å, °)

*D*—H⋯*A*	*D*—H	H⋯*A*	*D*⋯*A*	*D*—H⋯*A*
C16—H16⋯O1^i^	0.93	2.59	3.503 (2)	168
C6—H6⋯O3^ii^	0.93	2.62	3.420 (2)	144

Symmetry codes: (i) 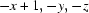; (ii) 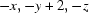.

**Fig. 1.**
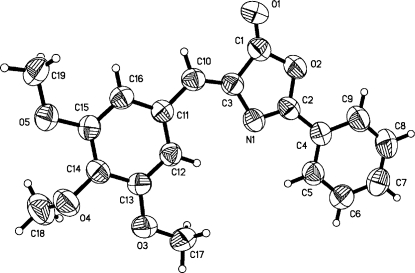
View of the title molecule showing the atom-labelling scheme. Displacement ellipsoids are drawn at the 50% probability level. H atoms are shown as small spheres of arbitrary radius.

## supplementary crystallographic information

### Comment

The development of highly efficient nonlinear optical crystals is extremely important for laser spectroscopy and laser processing. Oxazolone derivatives are highly versatile intermediates used for the synthesis of several biologically active organic molecules, such as amino acids, peptides, antimicrobial or antitumor compounds, immunomodulators, heterocyclic precursors for biosensor coupling, and photosensitive composition devices for proteins (Aaglawe *et al.*, 2003; Grassi *et al.*, 2004; Khan *et al.*, 2006). It has been reported (Song *et al.*, 2001) that some oxazolone derivatives exhibit promising nonlinear optical properties. The second-harmonic generation (SHG) value of the title compound is 1.821, as compared with urea powder. In this contribution, we report the crystal structure of the title compound.

The molecule possesses normal geometric parameters and adopts a *Z* configuration about the central olefinic bond (Fig. 1). The C11–C16 phenyl ring and the oxazolone ring are almost coplanar. However, the C4–C9 phenyl ring is slightly twisted out of the plane of the oxazolone ring system by 11.2 (2) °. Comparison with 2,6-dimethoxy-4- (5-oxo-2-phenyl-4,5-dihydro-1,3-oxazol-4-ylidenemethyl)-phenyl acetate (Sun *et al.*, 2007) suggests that the presence of the 4-methoxy group leads to this deviation from coplanarity. Also, while O3, O4, O5, C17 and C19 are approximately coplanar with their attached benzene ring, C18 deviate from their mother benzene ring (Fig. 1). The crystal structure is stabilized by the weak intermolecular C—H···O hydrogen bonds (Table 1).

Similar structures have been observed in the related oxazolone analogues reported by Sun *et al.* (2007), Imhof & Garms (2005), Song *et al.* (2004), and by Vasuki *et al.* (2001).

### Experimental

The title compound was synthesized from 3,4,5-trimethoxybenzaldehyde and hippuric acid as reported by Song *et al.* (2001). A mixture of hippuric acid (2.2 mmol), 3,4,5-trimethoxybenzaldehyde (2 mmol), sodium acetate (3 mmol) in acetic anhydride (8 ml) was refluxed for 5 hr. It was then cooled and ethanol (10 ml) was added to it. The resulting mixture was left over night at room temp. The solid thus obtained was filtered, dried and crystallized from ethanol to yield the title compound in 73% yield. A single-crystal suitable for an X-ray structural analysis was obtained by slowly evaporating from ethanol at room temperature.

### Refinement

All H atoms were initially located in a difference Fourier map. The methyl H atoms were then constrained to an ideal geometry with C—H distances of 0.96 Å and *U*_iso_(H) = 1.5*U*_eq_(C). All other H atoms were placed in geometrically idealized positions and constrained to ride on their parent atoms with C—H distances 0.93 Å and *U*_iso_(H) = 1.2*U*_eq_(C).

### Crystal data

**Table:**

C_19_H_17_NO_5_	*Z* = 2
*M**_r_* = 339.34	*F*_000_ = 356
Triclinic, *P*1	*D*_x_ = 1.358 Mg m^−^^3^
Hall symbol: -P 1	Mo *K*α radiation λ = 0.71073 Å
*a* = 7.3897 (5) Å	Cell parameters from 1358 reflections
*b* = 8.1532 (6) Å	θ = 2.9–24.7º
*c* = 14.0023 (9) Å	µ = 0.10 mm^−^^1^
α = 86.917 (5)º	*T* = 273 (2) K
β = 83.306 (4)º	Block, yellow
γ = 82.471 (5)º	0.15 × 0.12 × 0.10 mm
*V* = 830.02 (10) Å^3^	

### Data collection

**Table:**

Bruker SMART CCD area detector diffractometer	2904 independent reflections
Radiation source: fine-focus sealed tube	2056 reflections with *I* > 2σ(*I*)
Monochromator: graphite	*R*_int_ = 0.020
*T* = 273(2) K	θ_max_ = 25.1º
φ and ω scans	θ_min_ = 1.5º
Absorption correction: multi-scan(SADABS; Sheldrick, 1996)	*h* = −8→8
*T*_min_ = 0.985, *T*_max_ = 0.990	*k* = −9→8
4665 measured reflections	*l* = −16→14

### Refinement

**Table:**

Refinement on *F*^2^	Secondary atom site location: difference Fourier map
Least-squares matrix: full	Hydrogen site location: difference Fourier map
*R*[*F*^2^ > 2σ(*F*^2^)] = 0.043	H-atom parameters constrained
*wR*(*F*^2^) = 0.121	*w* = 1/[σ^2^(*F*_o_^2^) + (0.0602*P*)^2^ + 0.0668*P*] where *P* = (*F*_o_^2^ + 2*F*_c_^2^)/3
*S* = 1.04	(Δ/σ)_max_ < 0.001
2904 reflections	Δρ_max_ = 0.12 e Å^−^^3^
229 parameters	Δρ_min_ = −0.15 e Å^−^^3^
Primary atom site location: structure-invariant direct methods	Extinction correction: none

### Special details

**Table:**

Geometry. All e.s.d.'s (except the e.s.d. in the dihedral angle between two l.s. planes) are estimated using the full covariance matrix. The cell e.s.d.'s are taken into account individually in the estimation of e.s.d.'s in distances, angles and torsion angles; correlations between e.s.d.'s in cell parameters are only used when they are defined by crystal symmetry. An approximate (isotropic) treatment of cell e.s.d.'s is used for estimating e.s.d.'s involving l.s. planes.
Refinement. Refinement of *F*^2^ against ALL reflections. The weighted *R*-factor *wR* and goodness of fit *S* are based on *F*^2^, conventional *R*-factors *R* are based on *F*, with *F* set to zero for negative *F*^2^. The threshold expression of *F*^2^ > 2σ(*F*^2^) is used only for calculating *R*-factors(gt) *etc*. and is not relevant to the choice of reflections for refinement. *R*-factors based on *F*^2^ are statistically about twice as large as those based on *F*, and *R*- factors based on ALL data will be even larger.

### Fractional atomic coordinates and isotropic or equivalent isotropic displacement parameters (Å^2^)

**Table:**

	*x*	*y*	*z*	*U*_iso_*/*U*_eq_	
O1	0.5421 (2)	0.11697 (18)	−0.16116 (10)	0.0843 (5)	
O2	0.47613 (18)	0.36419 (17)	−0.23924 (9)	0.0666 (4)	
O3	−0.0038 (2)	0.77451 (16)	0.17976 (10)	0.0760 (4)	
O4	−0.0466 (2)	0.58784 (18)	0.33970 (10)	0.0759 (4)	
O5	0.09472 (19)	0.26856 (16)	0.34793 (9)	0.0685 (4)	
N1	0.3293 (2)	0.51906 (19)	−0.11774 (10)	0.0559 (4)	
C1	0.4742 (3)	0.2583 (3)	−0.15782 (14)	0.0626 (5)	
C2	0.3888 (2)	0.5149 (2)	−0.20767 (13)	0.0560 (5)	
C3	0.3759 (2)	0.3591 (2)	−0.07986 (13)	0.0546 (4)	
C4	0.3748 (2)	0.6523 (2)	−0.27805 (12)	0.0567 (5)	
C5	0.3176 (3)	0.8105 (3)	−0.24711 (14)	0.0656 (5)	
H5	0.2910	0.8276	−0.1816	0.079*	
C6	0.2996 (3)	0.9427 (3)	−0.31155 (15)	0.0720 (6)	
H6	0.2600	1.0486	−0.2898	0.086*	
C7	0.3402 (3)	0.9181 (3)	−0.40840 (15)	0.0764 (6)	
H7	0.3282	1.0074	−0.4524	0.092*	
C8	0.3981 (3)	0.7630 (3)	−0.43997 (15)	0.0836 (7)	
H8	0.4258	0.7471	−0.5056	0.100*	
C9	0.4161 (3)	0.6292 (3)	−0.37596 (14)	0.0742 (6)	
H9	0.4558	0.5237	−0.3983	0.089*	
C10	0.3399 (2)	0.2976 (2)	0.01042 (13)	0.0566 (5)	
H10	0.3867	0.1873	0.0207	0.068*	
C11	0.2390 (2)	0.3772 (2)	0.09430 (12)	0.0509 (4)	
C12	0.1654 (2)	0.5438 (2)	0.09198 (13)	0.0551 (4)	
H12	0.1786	0.6078	0.0352	0.066*	
C13	0.0733 (2)	0.6129 (2)	0.17411 (13)	0.0560 (4)	
C14	0.0515 (2)	0.5183 (2)	0.25978 (12)	0.0561 (5)	
C15	0.1226 (2)	0.3522 (2)	0.26160 (12)	0.0535 (4)	
C16	0.2174 (2)	0.2820 (2)	0.17932 (12)	0.0541 (4)	
H16	0.2666	0.1710	0.1810	0.065*	
C17	0.0240 (3)	0.8792 (2)	0.09656 (15)	0.0776 (6)	
H17A	0.1533	0.8797	0.0785	0.116*	
H17B	−0.0321	0.9897	0.1101	0.116*	
H17C	−0.0303	0.8391	0.0448	0.116*	
C18	0.0640 (4)	0.6488 (3)	0.40255 (16)	0.1007 (8)	
H18A	0.1511	0.5600	0.4234	0.151*	
H18B	−0.0125	0.6940	0.4575	0.151*	
H18C	0.1284	0.7337	0.3694	0.151*	
C19	0.1573 (3)	0.0968 (3)	0.35144 (15)	0.0724 (6)	
H19A	0.1064	0.0437	0.3030	0.109*	
H19B	0.1190	0.0500	0.4137	0.109*	
H19C	0.2889	0.0802	0.3399	0.109*	

### Atomic displacement parameters (Å^2^)

**Table:**

	*U*^11^	*U*^22^	*U*^33^	*U*^12^	*U*^13^	*U*^23^
O1	0.1024 (12)	0.0654 (10)	0.0810 (10)	0.0056 (8)	−0.0041 (8)	−0.0184 (8)
O2	0.0715 (8)	0.0709 (9)	0.0554 (7)	−0.0040 (7)	0.0000 (6)	−0.0128 (7)
O3	0.0942 (11)	0.0559 (8)	0.0699 (9)	0.0064 (7)	0.0051 (7)	0.0006 (7)
O4	0.0790 (9)	0.0789 (9)	0.0652 (8)	−0.0073 (7)	0.0123 (7)	−0.0120 (7)
O5	0.0813 (9)	0.0644 (8)	0.0575 (8)	−0.0123 (7)	0.0021 (6)	0.0062 (6)
N1	0.0542 (9)	0.0615 (10)	0.0526 (9)	−0.0109 (7)	−0.0033 (7)	−0.0048 (7)
C1	0.0636 (12)	0.0642 (13)	0.0608 (11)	−0.0072 (10)	−0.0074 (9)	−0.0108 (10)
C2	0.0492 (10)	0.0639 (12)	0.0564 (11)	−0.0098 (8)	−0.0043 (8)	−0.0124 (9)
C3	0.0531 (10)	0.0557 (11)	0.0561 (10)	−0.0084 (8)	−0.0068 (8)	−0.0067 (9)
C4	0.0490 (10)	0.0685 (12)	0.0537 (10)	−0.0114 (9)	−0.0047 (8)	−0.0052 (9)
C5	0.0624 (12)	0.0762 (14)	0.0563 (11)	−0.0055 (10)	−0.0022 (9)	−0.0031 (10)
C6	0.0673 (13)	0.0734 (14)	0.0740 (14)	−0.0078 (10)	−0.0058 (10)	0.0020 (11)
C7	0.0715 (13)	0.0897 (16)	0.0693 (13)	−0.0199 (12)	−0.0095 (11)	0.0132 (12)
C8	0.0985 (17)	0.0996 (18)	0.0541 (12)	−0.0228 (14)	−0.0034 (11)	−0.0001 (12)
C9	0.0891 (15)	0.0769 (14)	0.0570 (12)	−0.0157 (11)	−0.0004 (10)	−0.0088 (10)
C10	0.0578 (11)	0.0521 (10)	0.0613 (11)	−0.0089 (8)	−0.0086 (9)	−0.0049 (8)
C11	0.0502 (10)	0.0510 (10)	0.0530 (10)	−0.0107 (8)	−0.0063 (8)	−0.0042 (8)
C12	0.0585 (11)	0.0547 (11)	0.0520 (10)	−0.0098 (8)	−0.0044 (8)	0.0010 (8)
C13	0.0556 (10)	0.0509 (10)	0.0606 (11)	−0.0049 (8)	−0.0042 (9)	−0.0030 (9)
C14	0.0527 (10)	0.0600 (11)	0.0552 (10)	−0.0097 (8)	0.0014 (8)	−0.0076 (9)
C15	0.0521 (10)	0.0579 (11)	0.0519 (10)	−0.0137 (8)	−0.0054 (8)	0.0020 (8)
C16	0.0542 (10)	0.0495 (10)	0.0600 (11)	−0.0100 (8)	−0.0086 (8)	−0.0010 (8)
C17	0.0939 (16)	0.0551 (12)	0.0790 (14)	0.0018 (11)	−0.0067 (12)	0.0070 (10)
C18	0.146 (2)	0.0862 (17)	0.0716 (14)	−0.0273 (16)	0.0020 (15)	−0.0237 (12)
C19	0.0762 (13)	0.0681 (14)	0.0708 (13)	−0.0087 (11)	−0.0078 (11)	0.0161 (10)

### Geometric parameters (Å, °)

**Table:**

O1—C1	1.196 (2)	C8—C9	1.377 (3)
O2—C2	1.381 (2)	C8—H8	0.9300
O2—C1	1.393 (2)	C9—H9	0.9300
O3—C13	1.368 (2)	C10—C11	1.452 (2)
O3—C17	1.418 (2)	C10—H10	0.9300
O4—C14	1.368 (2)	C11—C16	1.391 (2)
O4—C18	1.418 (3)	C11—C12	1.396 (2)
O5—C15	1.363 (2)	C12—C13	1.374 (2)
O5—C19	1.417 (2)	C12—H12	0.9300
N1—C2	1.285 (2)	C13—C14	1.396 (3)
N1—C3	1.397 (2)	C14—C15	1.387 (3)
C1—C3	1.466 (3)	C15—C16	1.387 (2)
C2—C4	1.453 (3)	C16—H16	0.9300
C3—C10	1.345 (3)	C17—H17A	0.9600
C4—C5	1.380 (3)	C17—H17B	0.9600
C4—C9	1.387 (3)	C17—H17C	0.9600
C5—C6	1.371 (3)	C18—H18A	0.9600
C5—H5	0.9300	C18—H18B	0.9600
C6—C7	1.374 (3)	C18—H18C	0.9600
C6—H6	0.9300	C19—H19A	0.9600
C7—C8	1.361 (3)	C19—H19B	0.9600
C7—H7	0.9300	C19—H19C	0.9600
			
C2—O2—C1	105.35 (14)	C16—C11—C10	117.84 (16)
C13—O3—C17	117.23 (15)	C12—C11—C10	122.32 (16)
C14—O4—C18	113.61 (16)	C13—C12—C11	119.65 (16)
C15—O5—C19	117.33 (15)	C13—C12—H12	120.2
C2—N1—C3	105.67 (15)	C11—C12—H12	120.2
O1—C1—O2	121.82 (18)	O3—C13—C12	124.40 (16)
O1—C1—C3	133.22 (19)	O3—C13—C14	114.73 (16)
O2—C1—C3	104.96 (17)	C12—C13—C14	120.87 (17)
N1—C2—O2	115.89 (16)	O4—C14—C15	120.64 (16)
N1—C2—C4	126.61 (17)	O4—C14—C13	119.91 (17)
O2—C2—C4	117.50 (15)	C15—C14—C13	119.39 (16)
C10—C3—N1	129.17 (16)	O5—C15—C16	124.20 (16)
C10—C3—C1	122.71 (18)	O5—C15—C14	115.69 (16)
N1—C3—C1	108.10 (15)	C16—C15—C14	120.10 (16)
C5—C4—C9	118.72 (18)	C15—C16—C11	120.14 (16)
C5—C4—C2	119.35 (16)	C15—C16—H16	119.9
C9—C4—C2	121.92 (18)	C11—C16—H16	119.9
C6—C5—C4	120.99 (18)	O3—C17—H17A	109.5
C6—C5—H5	119.5	O3—C17—H17B	109.5
C4—C5—H5	119.5	H17A—C17—H17B	109.5
C5—C6—C7	119.7 (2)	O3—C17—H17C	109.5
C5—C6—H6	120.1	H17A—C17—H17C	109.5
C7—C6—H6	120.1	H17B—C17—H17C	109.5
C8—C7—C6	120.0 (2)	O4—C18—H18A	109.5
C8—C7—H7	120.0	O4—C18—H18B	109.5
C6—C7—H7	120.0	H18A—C18—H18B	109.5
C7—C8—C9	120.8 (2)	O4—C18—H18C	109.5
C7—C8—H8	119.6	H18A—C18—H18C	109.5
C9—C8—H8	119.6	H18B—C18—H18C	109.5
C8—C9—C4	119.8 (2)	O5—C19—H19A	109.5
C8—C9—H9	120.1	O5—C19—H19B	109.5
C4—C9—H9	120.1	H19A—C19—H19B	109.5
C3—C10—C11	129.81 (17)	O5—C19—H19C	109.5
C3—C10—H10	115.1	H19A—C19—H19C	109.5
C11—C10—H10	115.1	H19B—C19—H19C	109.5
C16—C11—C12	119.84 (16)		
			
C2—O2—C1—O1	−179.16 (18)	C1—C3—C10—C11	−177.22 (17)
C2—O2—C1—C3	1.44 (18)	C3—C10—C11—C16	178.06 (17)
C3—N1—C2—O2	−0.07 (19)	C3—C10—C11—C12	−2.3 (3)
C3—N1—C2—C4	−179.39 (16)	C16—C11—C12—C13	0.5 (3)
C1—O2—C2—N1	−0.9 (2)	C10—C11—C12—C13	−179.12 (16)
C1—O2—C2—C4	178.45 (15)	C17—O3—C13—C12	−4.2 (3)
C2—N1—C3—C10	−177.93 (18)	C17—O3—C13—C14	176.14 (17)
C2—N1—C3—C1	1.01 (18)	C11—C12—C13—O3	179.96 (16)
O1—C1—C3—C10	−1.8 (3)	C11—C12—C13—C14	−0.4 (3)
O2—C1—C3—C10	177.47 (16)	C18—O4—C14—C15	87.7 (2)
O1—C1—C3—N1	179.2 (2)	C18—O4—C14—C13	−95.3 (2)
O2—C1—C3—N1	−1.55 (19)	O3—C13—C14—O4	2.2 (2)
N1—C2—C4—C5	10.3 (3)	C12—C13—C14—O4	−177.52 (16)
O2—C2—C4—C5	−168.97 (16)	O3—C13—C14—C15	179.23 (15)
N1—C2—C4—C9	−169.19 (18)	C12—C13—C14—C15	−0.5 (3)
O2—C2—C4—C9	11.5 (3)	C19—O5—C15—C16	−4.0 (2)
C9—C4—C5—C6	0.8 (3)	C19—O5—C15—C14	176.91 (16)
C2—C4—C5—C6	−178.80 (17)	O4—C14—C15—O5	−2.7 (2)
C4—C5—C6—C7	−0.5 (3)	C13—C14—C15—O5	−179.76 (15)
C5—C6—C7—C8	0.0 (3)	O4—C14—C15—C16	178.17 (15)
C6—C7—C8—C9	0.2 (3)	C13—C14—C15—C16	1.1 (3)
C7—C8—C9—C4	0.0 (3)	O5—C15—C16—C11	180.00 (15)
C5—C4—C9—C8	−0.5 (3)	C14—C15—C16—C11	−1.0 (3)
C2—C4—C9—C8	179.02 (19)	C12—C11—C16—C15	0.1 (2)
N1—C3—C10—C11	1.6 (3)	C10—C11—C16—C15	179.81 (15)

### Hydrogen-bond geometry (Å, °)

**Table:**

*D*—H···*A*	*D*—H	H···*A*	*D*···*A*	*D*—H···*A*
C16—H16···O1^i^	0.93	2.59	3.503 (2)	168
C6—H6···O3^ii^	0.93	2.62	3.420 (2)	144

Symmetry codes: (i) −*x*+1, −*y*, −*z*; (ii) −*x*, −*y*+2, −*z*.
